# Epidemiology and First Molecular Characterization of *Sarcocystis* spp. in Roe Deer (*Capreolus capreolus*) from Western Romania with Phylogenetic Insights

**DOI:** 10.3390/ani16111681

**Published:** 2026-05-30

**Authors:** Florian Codrean, Tamás Szűts, Mirela Imre, Adriana Morar, Răzvan-Tudor Pătrînjan, Kálmán Imre

**Affiliations:** 1 Faculty of Veterinary Medicine, University of Life Sciences “King Mihai I” from Timisoara, 300645 Timisoara, Romania; codreanflorian97@gmail.com (F.C.); adrianamorar@usvt.ro (A.M.); razvan.patrinjan.fmv@usvt.ro (R.-T.P.); kalmanimre@usvt.ro (K.I.); 2 Diversity and Evolution Research Group, Department of Zoology, University of Veterinary Medicine Budapest, Rottenbiller utca 50, 1071 Budapest, Hungary; szuts.tamas@univet.hu

**Keywords:** protozoan parasites, molecular epidemiology, 18S rRNA, cervids, Romania

## Abstract

*Sarcocystis* spp. are common intracellular protozoan parasites of wild and domestic ungulates, yet data regarding their occurrence and molecular diversity in roe deer (*Capreolus capreolus*) from Romania remain scarce. The present study investigated the epidemiology and molecular diversity of *Sarcocystis* spp. in roe deer from western Romania and assessed the phylogenetic relationships of the detected isolates. A total of 132 striated muscle samples were collected from roe deer harvested in Arad, Bihor, and Timiș counties during 2023–2025 and examined microscopically for the presence of sarcocysts. Positive samples were subjected to PCR amplification and sequencing of the 18S rRNA gene. Sarcocysts were detected in 102/132 animals, yielding an overall prevalence of 77.3% (95% CI: 69.4–83.7). Infection prevalence increased significantly with host age (*p* < 0.001) and was significantly higher in males than in females (*p* < 0.05), whereas no significant differences were observed among counties or sampling years (*p* > 0.05). Molecular analysis of 30 representative isolates identified three *Sarcocystis* species: *Sarcocystis gracilis* (46.7%), *S. linearis* (33.3%), and *S. entzerothi* (20.0%), with *S. gracilis* being the predominant species. Phylogenetic analysis based on partial 18S rRNA sequences confirmed species-level identification and demonstrated close clustering of Romanian isolates with homologous European reference sequences, indicating high genetic similarity and limited phylogeographic structuring. To the authors’ knowledge, this study represents the first molecular characterization of *Sarcocystis* spp. in roe deer from Romania and expands current knowledge on the epidemiology, species diversity, and phylogenetic relationships of roe deer-associated *Sarcocystis* in Europe.

## Simple Summary

This study investigated the occurrence and genetic diversity of *Sarcocystis* parasites in roe deer (*Capreolus capreolus*) from western Romania. Muscle samples collected between 2023 and 2025 showed a high infection rate (77.3%), with prevalence increasing with age and being higher in males. Molecular analyses identified three species, namely *Sarcocystis gracilis*, *S. linearis*, and *S. entzerothi*, with *S. gracilis* being the most common. Phylogenetic results revealed that Romanian isolates are closely related to other European strains, suggesting low genetic variation across regions. This study provides the first molecular data on *Sarcocystis* in roe deer from Romania and contributes to understanding their epidemiology and diversity in Europe.

## 1. Introduction

Species of the genus *Sarcocystis* are intracellular protozoan parasites (Apicomplexa) with an obligatory two-host life cycle, involving a prey–predator relationship between intermediate and definitive hosts [1,2]. Herbivorous mammals, including wild cervids, act as intermediate hosts in which sarcocysts develop within striated muscles, while carnivores serve as definitive hosts, shedding sporocysts into the environment [3,4,5]. Infections are typically subclinical [6]. However, heavy parasitism may lead to clinical disease, including reduced fitness, reproductive disorders, and, in severe cases, mortality, thus highlighting their potential impact on wildlife health and ecosystem dynamics [7,8].

Beyond their importance in wildlife ecology, *Sarcocystis* spp. are increasingly recognized within a One Health framework, due to their highly adapted transmission cycles involving wildlife, domestic animals, and humans [9]. The One Health approach recognizes the interconnectedness between human, animal, and environmental health, emphasizing that pathogens circulating in wildlife may also affect domestic animals and humans through shared ecosystems and transmission pathways. Environmental contamination with sporocysts shed by definitive hosts such as canids, corvids and birds of prey may facilitate the spread of infection across ecosystems, including agricultural settings and water sources [10]. Consequently, these parasites may contribute to infection pressure in domestic livestock (e.g., cattle, sheep, and pigs), with potential economic and animal health implications [11]. Furthermore, certain *Sarcocystis* species are of zoonotic relevance, as humans may become infected either as definitive hosts through consumption of raw or undercooked meat containing sarcocysts or as accidental hosts via ingestion of contaminated food or water [8]. Although the zoonotic role of cervid-associated *Sarcocystis* species remains incompletely understood, the increasing consumption of game meat, including venison, highlights the need to better characterize their potential public health significance [10,11].

The European roe deer (*Capreolus capreolus*) is one of the most widespread and abundant wild ungulates in Europe and represents an important reservoir host for a wide range of parasitic infections [8]. Among these, *Sarcocystis* spp. are frequently reported, with prevalence values often exceeding 70% and, in some regions, approaching 100% [1,3,4,5,7]. Molecular and morphological investigations have demonstrated that roe deer can harbor at least six *Sarcocystis* species, including *S. gracilis*, *S. linearis*, *S. entzerothi*, *S. capreolicanis*, *S. oviformis*, and *S. silva*, with considerable variation in species composition across geographic regions [8]. As prey species for wild and domestic carnivores (including dogs), roe deer may play a key role in maintaining transmission cycles at the wildlife–domestic interface.

Over the past decades, advances in molecular techniques, particularly PCR amplification and sequencing of markers such as the 18S rRNA and *cox1* genes, have significantly improved the identification and phylogenetic characterization of *Sarcocystis* species [9]. These approaches have revealed high genetic similarity among isolates from different European regions [6,10], but also highlighted intra- and interspecific variability, as well as the presence of cryptic or newly described species [7,12]. Furthermore, phylogenetic analyses have contributed to a better understanding of host specificity, evolutionary relationships, and transmission patterns within the genus [4,5,12], which are essential for assessing their epidemiological and potential zoonotic significance.

Despite the growing body of research across Europe, including studies conducted in countries such as Italy, Lithuania, Spain, Poland, and Norway [1,3,4,5,7,12], data regarding the epidemiology and molecular diversity of *Sarcocystis* spp. in roe deer remain limited or completely lacking in certain regions [8]. In Romania, although roe deer populations are widespread and ecologically important, information on *Sarcocystis* infection in this host is scarce. Only a small study has been conducted reporting preliminary data on the presence or absence of the parasite in a limited number of animals [13], and no comprehensive molecular characterization has been reported to date. The epidemiology of *Sarcocystis* spp. in wild ungulates may also reflect the broader circulation of canid-associated apicomplexan parasites within the same ecosystems. Previous studies conducted in Western Romania demonstrated exposure of domestic animals to *Neospora caninum* [14], supporting the presence of ecological conditions favorable for the maintenance of canid-transmitted protozoan parasites in the region.

Therefore, the present study aimed to determine the prevalence of *Sarcocystis* infection in roe deer (*Capreolus capreolus*) from western Romania and to provide the first molecular characterization of circulating species based on 18S rRNA gene analysis. In addition, phylogenetic analyses were performed to elucidate the genetic relationships of the identified isolates in a broader European context.

## 2. Materials and Methods

### 2.1. Sample Collection

**Figure 1:**
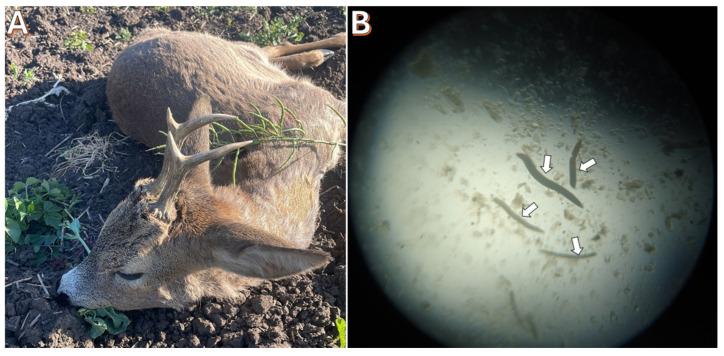
(**A**) Hunted roe deer (*Capreolus capreolus*) carcass during field sampling in Western Romania. (**B**) Microscopic appearance of sarcocysts (Miescher’s tubules), indicated by narrows, detected in roe deer skeletal muscle samples during parasitological examination.

This study was conducted over a period of three consecutive years (2023–2025), during the legally designated hunting season for roe deer (May 1 to October 15 in each year). Sampling was carried out across eleven hunting grounds located in Western Romania, distributed within the counties of Arad (*n* = 5), Bihor (*n* = 3), and Timiș (*n* = 3). A total of 132 samples were collected, consisting of striated muscle tissue obtained from the hip region (approximately 200 g per sample) from roe deer (*Capreolus capreolus*) (Figure 1A), harvested by shooting during organized hunting activities conducted by hunting associations. Although heart and diaphragm are commonly preferred for *Sarcocystis* detection due to their high cyst density in cervids [12,15], hip musculature was intentionally selected in the present study because it represents edible tissue commonly destined for human consumption, thereby being more relevant from a food safety and zoonotic surveillance perspective [16]. In contrast, the organ package and less edible tissues such as the diaphragm are frequently discarded during hunting practices. Nevertheless, sarcocysts are known to occur broadly in skeletal musculature of intermediate hosts, including multiple striated muscle groups [13], supporting the suitability of hip musculature for epidemiological screening. Specifically, samples were collected from the gluteal musculature of the hip region.

During sample collection, individual animal (gender and age) and epidemiological data (location of hunting ground) were provided by the hunters. The collected samples were transported under refrigerated conditions (0–4 °C) to the laboratory, where they were stored frozen (−20 °C) until processing, which occurred between 24 h and 21 days post-collection.

### 2.2. Microscopy and Molecular Analyses

On the day of processing, the muscle tissue samples were gradually thawed under refrigeration conditions. Subsequently, a total of 10 g of striated muscle tissue from each animal was processed for the detection of sarcocysts (Miescher’s tubules) (Figure 1B) using the fresh preparation isolation method, according to the technique described by Moré et al. [16]. Briefly, the protocol involved weighing 10 g of tissue, followed by mechanical homogenization using a blender. The homogenized material was then mixed with 100 mL of tap water and subjected to continuous stirring on a magnetic stirrer for 30 min. The parasite isolation was achieved by sedimentation, which included filtration of the homogenate through a 2 mm mesh sieve, followed by a resting period of 15 min in a separatory funnel equipped with a metal clamp. The presence of sarcocysts was assessed by using a stereomicroscope at 10–40× magnification. For each positive sample, between 5 and 10 individual sarcocysts were selected, repeatedly washed with cold water, and subsequently stored in 50 µL distilled water and stored at −20 °C until further molecular analyses, aiming to identify the involved *Sarcocystis* spp. [17,18].

Genomic DNA was extracted from the isolated sarcocysts using a commercial extraction kit (Isolate Genomic DNA Kit, Bioline Reagents Limited^®^, London, UK), following the protocol recommended by the manufacturer. The obtained DNA served as a template for genus-specific polymerase chain reaction (PCR) assays targeting a fragment of the 18S ribosomal RNA gene, approximately 915 bp in length. Amplification was carried out under conditions previously reported by Yang et al. [19], employing the primer pair 2L forward (5′-GGATAAACCGTGGTAATTCTATG-3′) and 3H reverse (5′-GGCAAATGCTTTCGCAGTAG-3′). Each PCR run included both positive and negative controls to ensure the reliability and accuracy of the amplification process.

The resulting PCR products were separated by electrophoresis on 2.2% agarose gels and stained with Midori Green™ (Nippon Genetics^®^, Europe GmbH, Düren, Germany) to allow visualization under UV illumination. For species determination, a subset of 30 representative amplicons was selected using a stratified sampling design [15], purified, and subjected to sequencing (Macrogen Europe^®^, Amsterdam, The Netherlands) using the same primer set. The selection included 9 isolates from Arad, 12 from Bihor, and 9 from Timiș counties. All available calf samples (*n* = 5) were included, alongside 10 yearlings and 15 adults. Sex distribution was maintained proportionally (20 females and 10 males) to reflect the original dataset.

The obtained nucleotide sequences were subsequently edited and aligned using Clustal X software version 2.1, followed by comparative analysis with reference sequences available in the GenBank™ database. Sequence similarity and species identification were determined by BLAST analysis version 2.17.0 (National Center for Biotechnology Information, available online: https://blast.ncbi.nlm.nih.gov/Blast.cgi, accessed on 27 May 2026). A single representative sequence for each species was selected and deposited in GenBank^TM^ under the accession numbers: PZ054912 (*S. gracilis*), PZ059403 (*S. linearis*), and PZ054641 (*S. entzerothi*).

### 2.3. Phylogenetic Analyses

For the phylogenetic analysis, representative 18S rRNA gene sequences of *Sarcocystis* spp., available in GenBank^TM^, were selected to ensure a comprehensive comparison with the isolates obtained in the present study. The dataset included sequences of all *Sarcocystis* species previously reported from roe deer (*Capreolus capreolus*), namely *Sarcocystis gracilis*, *S. silva*, *S. capreolicanis*, *S. oviformis*, *S. entzerothi*, and *S. linearis*, originating from several European countries. In order to provide a broader phylogenetic context, additional *Sarcocystis* sequences isolated from other cervid hosts, including red deer (*Cervus elaphus*), reindeer (*Rangifer tarandus*), and moose (*Alces alces*), were also included. Furthermore, sequences of *Sarcocystis* species infecting domestic animals in Romania, such as *S. cruzi* from cattle (*Bos taurus*) and *S. miescheriana* from pigs (*Sus scrofa domesticus*), were incorporated to provide reference points within the genus. The apicomplexan species, *Neospora caninum*, was used as an outgroup to root the phylogenetic tree. In total, the dataset comprised 38 reference sequences retrieved from GenBank^TM^ together with the sequences generated in this study.

To verify the *Sarcocystis* species placements, a phylogenetic analysis of the 18S rRNA gene was performed. To provide a robust phylogenetic background for our diagnostic sequences, which were ~600 bp long, we selected sequences from GenBank^TM^ that were over 1500 bp long. The sequences were aligned using MAFFT [20] and trimmed in BGME [21], with the final alignment being 1818 nucleotides long. Model selection was performed in SMS [22], resulting in the TN93 G+I substitution model, and the maximum likelihood tree was inferred in PhyML [23] on the NGPhylogeny server [24]. Bootstrap values were calculated according to the method of Lemoine et al. (2018) [25], and the final tree was edited in iTol [26].

### 2.4. Statistical Analyses

Prevalence was calculated with 95% confidence intervals (95% CI) using the Wilson method. Associations between infection status and categorical variables (county, age, sex, and year) were assessed using the chi-square (χ^2^) test, while Fisher’s exact test was applied when expected frequencies were low. For the molecular data (*n* = 30), species proportions were compared using a chi-square goodness-of-fit test, and associations with host and epidemiological variables were evaluated using Fisher’s exact test. Differences were considered statistically significant at *p* < 0.05.

## 3. Results

Sarcocysts were detected in 102 of 132 examined roe deer, corresponding to an overall prevalence of 77.3% (95% CI: 69.4–83.7). Infection was identified in roe deer from all investigated counties, with no significant geographic differences in prevalence (χ^2^ test, *p* > 0.05; Table 1). Prevalence increased with host age (χ^2^ test, *p* < 0.001), with the most evident difference observed between calves and adults, while differences involving yearlings should be interpreted cautiously due to the limited number of calves included in the study. Males were significantly more frequently infected than females (χ^2^ test, *p* < 0.05). Although prevalence varied moderately among years, no significant temporal differences were observed (χ^2^ test, *p* > 0.05).

A subset of 30 isolates was analyzed by PCR amplification and sequencing of the 18S rRNA gene. Molecular characterization identified three *Sarcocystis* species: *S. gracilis*, *S. linearis*, and *S. entzerothi*. The predominant species was *S. gracilis* (46.7%; 14/30), followed by *S. linearis* (33.3%; 10/30) and *S. entzerothi* (20.0%; 6/30), although these differences were not statistically significant (χ^2^ goodness-of-fit test, *p* > 0.05).

All three species were detected in Arad and Bihor, whereas only *S. gracilis* and *S. linearis* were identified in Timiș. No geographic differences in species distribution were observed (Fisher’s exact test, *p* > 0.05). The species *S. gracilis* predominated in all counties. All three species occurred in yearlings and adults, while calves harbored only *S. gracilis* and *S. linearis*. Although *S. entzerothi* was detected only in older animals, this finding should be interpreted cautiously, as mixed *Sarcocystis* infections may occur in roe deer and the applied PCR/sequencing approach may preferentially detect the predominant species present in the sample. Likewise, species distribution did not differ significantly between sexes (Fisher’s exact test, *p* > 0.05), despite *S. gracilis* being more frequent in females. Species diversity was highest in 2024, when all three species were detected, but no significant temporal differences in species distribution were observed among study years (Fisher’s exact test, *p* > 0.05).

**Figure 2:**
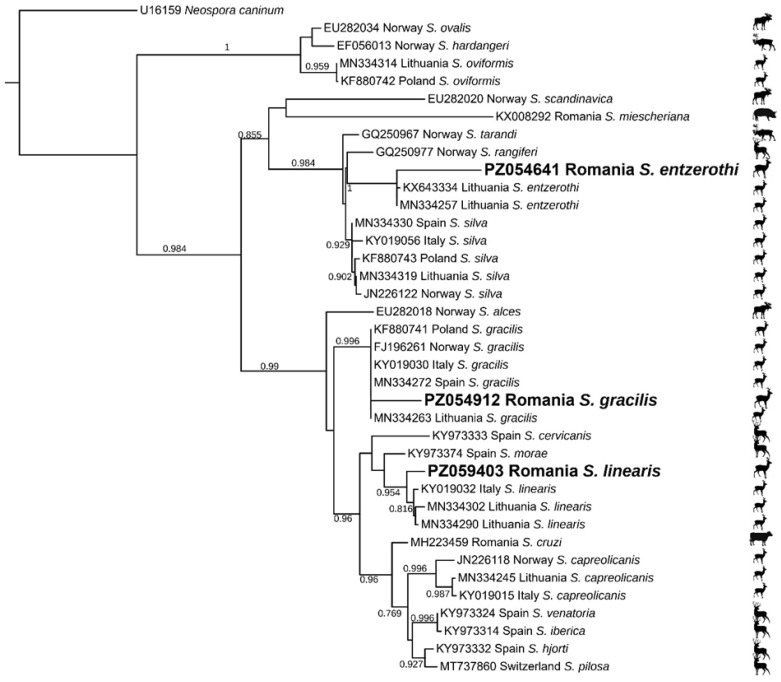
Maximum-likelihood phylogenetic tree based on partial 18S rRNA gene sequences (~600 bp) showing the relationships between *Sarcocystis* isolates identified in roe deer (*Capreolus capreolus*) from Western Romania and reference *Sarcocystis* spp. sequences retrieved from GenBank^TM^. Bootstrap support values are indicated at the nodes. Sequences generated in the present study are highlighted in bold. *Neospora caninum* was used as the outgroup.

Phylogenetic analysis of partial 18S rRNA gene sequences confirmed the species-level identification of the Romanian isolates and demonstrated their clustering within well-supported clades corresponding to recognized *Sarcocystis* species (Figure 2).

The Romanian *S. gracilis* isolate (PZ054912) grouped within the *S. gracilis* clade together with reference sequences from Poland, Norway, Italy, Spain, and Lithuania, showing close phylogenetic affinity to other European isolates. Similarly, the Romanian *S. linearis* isolate (PZ059403) clustered within the *S. linearis* clade alongside sequences from Italy and Lithuania, forming a strongly supported monophyletic group. The Romanian *S. entzerothi* isolate (PZ054641) grouped with previously reported *S. entzerothi* sequences from Lithuania, confirming its taxonomic assignment and close genetic relationship with isolates circulating in other European roe deer populations. Overall, the phylogenetic topology demonstrated high genetic similarity between the Romanian isolates and previously characterized European *Sarcocystis* spp., with no evidence of marked geographic segregation among isolates.

## 4. Discussion

The present study provides the first molecular characterization of *Sarcocystis* spp. infecting roe deer (*Capreolus capreolus*) in Romania and expands the limited epidemiological and molecular data currently available from Eastern and Southeastern Europe. The overall prevalence of sarcocyst infection detected in the present study (77.3%) confirms that roe deer in western Romania are commonly infected with *Sarcocystis* spp., corroborating the preliminary observations previously reported in the same geographical region [13]. This prevalence is consistent with the generally high infection rates reported in roe deer throughout Europe, where *Sarcocystis* prevalence frequently exceeds 70% and may approach 100% depending on the detection method, muscle type examined, and geographical area [1,3,4,5,7,8,12]. Since only the hip striated muscle was examined, the true prevalence and species diversity may have been underestimated compared with studies using predilection sites such as the heart and diaphragm [15]. However, hip musculature was intentionally selected because it represents edible tissue commonly destined for human consumption and was therefore considered relevant from a food safety and zoonotic surveillance perspective.

The widespread detection of infection across all investigated counties, together with the absence of significant geographic differences, suggests a homogeneous circulation of *Sarcocystis* spp. in roe deer populations throughout western Romania. Similar epidemiological patterns have been described in other European countries, where broad environmental contamination with sporocysts and the extensive distribution of suitable definitive hosts likely facilitate parasite transmission across large areas [8,11,15]. From a One Health perspective, such widespread environmental contamination is particularly relevant, as sporocysts shed by wild and domestic carnivores (including dogs and foxes) may persist in the environment and contaminate soil and water sources, thereby contributing to indirect exposure of both livestock and humans [27].

The significant age-related increase in prevalence observed in the present study, with adults showing the highest infection rates, is in agreement with the cumulative exposure pattern typically reported for *Sarcocystis* infections in wild ungulates and reflects progressive acquisition of infection over time [28,29,30]. This pattern further supports the notion of continuous environmental exposure to infective sporocysts, which may also have implications for domestic grazing animals sharing the same habitats or water sources [27].

The significantly higher prevalence recorded in males compared with females may indicate sex-related differences in habitat use, ranging behavior, or exposure to contaminated environments. Although sex-associated differences in *Sarcocystis* prevalence have not been consistently reported in roe deer [15], similar patterns have occasionally been observed in wildlife parasitology studies and may reflect behavioral rather than physiological determinants of exposure [31,32].

Molecular analysis identified three *Sarcocystis* species, namely *S. gracilis*, *S. linearis*, and *S. entzerothi* in Romanian roe deer. These species are among the six *Sarcocystis* taxa currently recognized in this host and have been previously reported from roe deer in other European countries, including Lithuania [7], Italy [4], Norway [1], and Spain [7]. The predominance of *S. gracilis* observed in the present study agrees with several previous investigations in European roe deer [8], in which this species has consistently been among the most frequently detected taxa. However, the detection of only three species in the present study, compared with the higher diversity reported in Lithuania (six species) and similar diversity observed in Spain (three species) [7], should be interpreted with caution, as methodological differences including tissue selection and the limited number of sarcocysts subjected to molecular characterization may have underestimated mixed infections and consequently the true species diversity present in Romanian roe deer populations.

Although the *Sarcocystis* species identified in roe deer are generally considered host-specific and of limited confirmed zoonotic relevance, their presence in edible tissues should not be overlooked. Game meat, including roe deer venison, is increasingly consumed in Europe, often through informal or local processing chains where veterinary inspection and adequate cooking practices may vary. In this context, the detection of a high prevalence of sarcocysts in muscle tissue highlights a potential, albeit currently underexplored, food safety concern. While human intestinal sarcocystosis is primarily associated with zoonotic species such as *S. hominis* and *S. suihominis* transmitted through the consumption of raw or undercooked beef and pork, the potential food safety relevance of cervid-associated *Sarcocystis* spp. remains insufficiently investigated. Considering the increasing consumption of wild ungulate meat in Europe, further studies are warranted to clarify the zoonotic potential of these parasites, particularly within a precautionary One Health framework, although direct evidence linking human sarcocystosis to consumption of infected cervid meat is currently limited [8,27].

Phylogenetic analysis demonstrated that the Romanian *Sarcocystis* isolates clustered consistently within the corresponding *S. gracilis*, *S. linearis*, and *S. entzerothi* clades and showed close affinity to homologous reference sequences from other European countries. This pattern indicates a high degree of genetic conservation among European roe deer-derived *Sarcocystis* isolates and corroborates previous molecular studies reporting limited sequence divergence within these taxa across broad geographic areas [7,12,33]. The absence of clear geographic structuring among isolates suggests that the 18S rRNA gene is highly conserved within roe deer-associated *Sarcocystis* species and may therefore have limited resolution for detecting fine-scale intraspecific variation, despite its suitability for species-level identification. Similar observations have been made in previous studies, in which isolates from geographically distant European populations clustered together with minimal divergence based on 18S rRNA sequences [1,4,5,7].

The close phylogenetic relationship between Romanian and other European isolates may reflect the broad distribution and ecological continuity of both roe deer populations and their carnivorous definitive hosts across Europe, facilitating parasite dispersal and gene flow. In particular, the role of wild carnivores such as foxes, as well as domestic dogs, in maintaining transmission cycles is likely to be of epidemiological importance, as these hosts can bridge wildlife and human-associated environments [11]. In addition, the relatively conserved nature of the 18S rRNA locus suggests that more variable genetic markers, such as *cox1* or ITS1, may be required in future studies to better resolve population-level diversity and potential regional structuring within roe deer-associated *Sarcocystis* spp. [7,12].

Several limitations of the present study should be acknowledged. Molecular characterization was performed on only a subset of positive samples, and therefore, the full diversity of *Sarcocystis* spp. circulating in Romanian roe deer may have been underestimated. In addition, only striated muscle from the hip region was examined, although some *Sarcocystis* species may exhibit tissue predilection, potentially affecting species detection. Furthermore, because only 5–10 individual sarcocysts were selected from each positive sample for molecular characterization, mixed infections involving multiple *Sarcocystis* species may have been underestimated or missed entirely, potentially contributing to the relatively low species diversity observed in the present study, as co-infections have previously been reported in cervids. In addition, only striated muscle from the hip region was examined, although some *Sarcocystis* species may exhibit tissue predilection, potentially affecting species detection. Moreover, species identification and phylogenetic inference were based exclusively on partial 18S rRNA gene sequences, a marker suitable for species-level discrimination but with limited resolution for assessing intraspecific variability and fine-scale phylogeographic structure. Future studies should incorporate larger sample sizes, multiple tissue types, and additional molecular markers such as *cox1* and ITS1 to better characterize the diversity and population structure of roe deer-associated *Sarcocystis* spp. in Romania.

From a broader perspective, future research should also address the role of definitive hosts, environmental contamination pathways (including water sources), and the potential transmission to domestic animals and humans [34]. Such integrated investigations would contribute to a more comprehensive understanding of *Sarcocystis* epidemiology within a One Health framework, linking wildlife reservoirs, livestock exposure, and public health considerations [27,35].

## 5. Conclusions

The present study provides the first molecular characterization of *Sarcocystis* spp. infecting roe deer (*Capreolus capreolus*) in Romania and demonstrates that sarcocyst infection is widespread in roe deer populations from western Romania. Three species, namely *Sarcocystis gracilis*, *S. linearis*, and *S. entzerothi*, were identified, with *S. gracilis* representing the predominant taxon. Phylogenetic analysis confirmed close genetic relationships between Romanian isolates and previously reported European strains, supporting the high genetic conservation of roe deer-associated *Sarcocystis* spp. across Europe. Beyond their relevance in wildlife parasitology, these findings highlight the potential role of roe deer as reservoirs contributing to the maintenance of *Sarcocystis* transmission cycles at the wildlife–livestock interface. The high prevalence of infection in edible tissues also underscores the need to consider game meat, including venison, within a broader food safety and public health context, particularly in regions where consumption and informal processing are common. Further studies focusing on the identification of definitive hosts, environmental persistence of sporocysts (including in water sources), and the potential zoonotic implications of cervid-associated *Sarcocystis* species are warranted.

Overall, the present study provides a baseline for future research on *Sarcocystis* epidemiology in Romania and contributes to a better understanding of parasite transmission dynamics within interconnected wildlife, livestock, and human systems.

## Figures and Tables

**Table 1:** Epidemiological characteristics, prevalence of *Sarcocystis* spp. infection, and distribution of molecularly identified *Sarcocystis* species in roe deer (*Capreolus capreolus*) from Western Romania according to county, age, sex, and year of sampling.

Individual Animal and Epidemiological Data	Positive/Total (% [CI95%])	Identified Species (Number)
**Counties**		
Arad	32/42 (76.2 [61.5–86.5])	*S. gracilis* (4), *S. linearis* (1), *S. entzerothi* (4)
Bihor	41/56 (73.2 [60.4–83.0])	*S. gracilis* (4), *S. linearis* (6), *S. entzerothi* (2)
Timiș	29/36 (80.6 [65.0–90.3])	*S. gracilis* (6), *S. linearis* (3)
**Age**		
Calf (<1 year)	5/12 (41.7 [19.3–68.1])	*S. gracilis* (3), *S. linearis* (2),
Yearling (1–2 years)	31/46 (67.4 [52.9–79.1])	*S. gracilis* (6), *S. linearis* (2), *S. entzerothi* (2)
Adult (>2 years)	66/76 (86.8 [77.4–92.7])	*S. gracilis* (5), *S. linearis* (6), *S. entzerothi* (4)
**Sex**		
Female	56/81 (69.1 [58.4–78.1])	*S. gracilis* (11), *S. linearis* (5), *S. entzerothi* (4)
Male	46/53 (86.8 [75.2–93.4])	*S. gracilis* (3), *S. linearis* (5), *S. entzerothi* (2)
**Year**		
2023	19/28 (67.8 [49.3–82.1])	*S. gracilis* (3), *S. linearis* (3)
2024	61/74 (82.4 [72.2–89.4])	*S. gracilis* (8), *S. linearis* (5), *S. entzerothi* (4)
2025	22/30 (73.3 [55.5–85.8])	*S. gracilis* (3), *S. linearis* (3), *S. entzerothi* (1)

## Data Availability

The data are contained within the article.

## Abbreviations

The following abbreviations are used in this manuscript:

bp	base pairs
CI	confidence interval
*cox1*	cytochrome c oxidase subunit I
DNA	deoxyribonucleic acid
ITS1	internal transcribed spacer 1
PCR	polymerase chain reaction
rRNA	ribosomal RNA
UV	ultraviolet
